# Anticancer efficacy of a nitric oxide‐modified derivative of bifendate against multidrug‐resistant cancer cells

**DOI:** 10.1111/jcmm.12796

**Published:** 2016-02-10

**Authors:** Zhiguang Ren, Xiaoke Gu, Bin Lu, Yaqiong Chen, Guojiang Chen, Jiannan Feng, Jizhen Lin, Yihua Zhang, Hui Peng

**Affiliations:** ^1^ Department of Environment and PharmacyTianjin Institute of Health and Environmental MedicineBeijingChina; ^2^ Department of ImmunologyInstitute of Basic Medical SciencesBeijingChina; ^3^ Jiangsu Key Laboratory of New Drug Research and Clinical PharmacyXuzhou Medical CollegeXuzhouChina; ^4^ State Key Laboratory of Natural MedicinesChina Pharmaceutical UniversityNanjingChina; ^5^ School of Laboratory Medicine and Life ScienceWenzhou Medical CollegeWenzhouChina; ^6^ Department of PharmacologyChina Pharmaceutical UniversityNanjingChina; ^7^ Department of OtolaryngologyUniversity of Minnesota Medical SchoolMinneapolisMNUSA

**Keywords:** multidrug resistance, nitric oxide, bifendate, anticancer

## Abstract

The development of multidrug resistance (MDR) not only actively transports a wide range of cytotoxic drugs across drug transporters but is also a complex interaction between a number of important cellular signalling pathways. Nitric oxide donors appear to be a new class of anticancer therapeutics for satisfying all the above conditions. Previously, we reported furoxan‐based nitric oxide‐releasing compounds that exhibited selective antitumour activity *in vitro* and *in vivo*. Herein, we demonstrate that bifendate (DDB)‐nitric oxide, a synthetic furoxan‐based nitric oxide‐releasing derivative of bifendate, effectively inhibits the both sensitive and MDR tumour cell viability at a comparatively low concentration. Interestingly, the potency of DDB‐nitric oxide is the independent of inhibition of the functions and expressions of three major ABC transporters. The mechanism of DDB‐nitric oxide appears to be in two modes of actions by inducing mitochondrial tyrosine nitration and apoptosis, as well as by down‐regulating HIF‐1α expression and protein kinase B (AKT), extracellular signal‐regulated kinases (ERK), nuclear factor κB (NF‐κB) activation in MDR cells. Moreover, the addition of a typical nitric oxide scavenger significantly attenuated all the effects of DDB‐nitric oxide, indicating that the cytotoxicity of DDB‐nitric oxide is as a result of higher levels of nitric oxide release in MDR cancer cells. Given that acquired MDR to nitric oxide donors is reportedly difficult to achieve and genetically unstable, compound like DDB‐nitric oxide may be a new type of therapeutic agent for the treatment of MDR tumours.

## Introduction

Multidrug resistance (MDR) in cancer has become a serious obstacle to successful clinical cancer chemotherapy. Multidrug resistance is characterized by decreased cellular sensitivities to a broad range of chemotherapeutic agents due, at least mostly, to the overexpression of efflux transport protein on the plasma membrane of cancer cells. Three major human ATP‐binding cassette (ABC) transporters have been specialized to play a key role in the clinical development of MDR, including P‐glycoprotein (P‐gp/ABCB1), breast cancer resistance protein (BCRP/ABCG2) and the multidrug resistance‐associated protein 1 (MRP1/ABCC1) 1, 2. In this regard, considerable attempts have been made to reverse MDR in tumours by developing inhibitors which target MDR‐related ABC (MDR‐ABC) transporters during the past decades. However, until now, none of them has been approved clinically for many reasons, such as low selectivity, poor potency, inherent toxicity and/or adverse pharmacokinetic interaction with co‐administered anticancer drugs 3. Therefore, development of new therapeutic agents that can directly kill both drug‐sensitive and ‐resistant tumour cells with less toxicity to normal cells is of great interest.

DDB‐nitric oxide is a synthetic nitric oxide‐releasing compound, which was synthesized by coupling bifendate (DDB) with nitric oxide‐donor furoxan through a chemical linker. The design and synthesis of DDB‐nitric oxide is mainly based on the following considerations. Firstly, as a synthetic liver protective drug, DDB has been clinically used for the treatment of viral hepatitis type B for more than 20 years in China with little side effects. Previous reports have documented that DDB is able to reverse ABCB1‐mediated MDR *in vitro* and *in vivo* by increasing intracellular accumulation of anticancer drugs and promoting the apoptosis through the inhibition of ABCB1 4. Further study revealed that six‐alkoxyl biphenyl skeleton in DDB is essential for its pharmacological activity 5. Therefore, its anticancer and MDR reversal activities together with low toxicity have made DDB one of the attractive lead compounds for our study. Secondly, nitric oxide, acting as a signalling and/or effector molecule, plays an important role in various physiological and pathological processes. Many reports have showed that high levels of nitric oxide derived from nitric oxide donors not only can inhibit the proliferation of tumour cells and induce the apoptosis of sensitive tumour cells but also can sensitize resistant tumour cells to chemotherapy, radiotherapy and immunotherapy *in vitro* and *in vivo* 6, 7. The underlying mechanisms of the action of nitric oxide include the inhibition of key transcription factors such as NF‐κB and HIF‐1α, as well as the inhibition of DNA‐repairing enzymes and drug efflux transporters, *etc*. Thirdly, it has been reported that acquired drug resistance induced by nitric oxide‐donors is hard to achieve and unstable genetically 8. Fourthly, our group has previously developed a variety of furoxan‐based nitric oxide‐releasing compounds that exhibited the selective antitumour activity *in vitro* and *in vivo* 9, 10. These findings indicate that furoxan/DDB hybrids are promising agents for anticancer therapeutics, especially for the treatment of MDR tumours.

**Figure 1:**
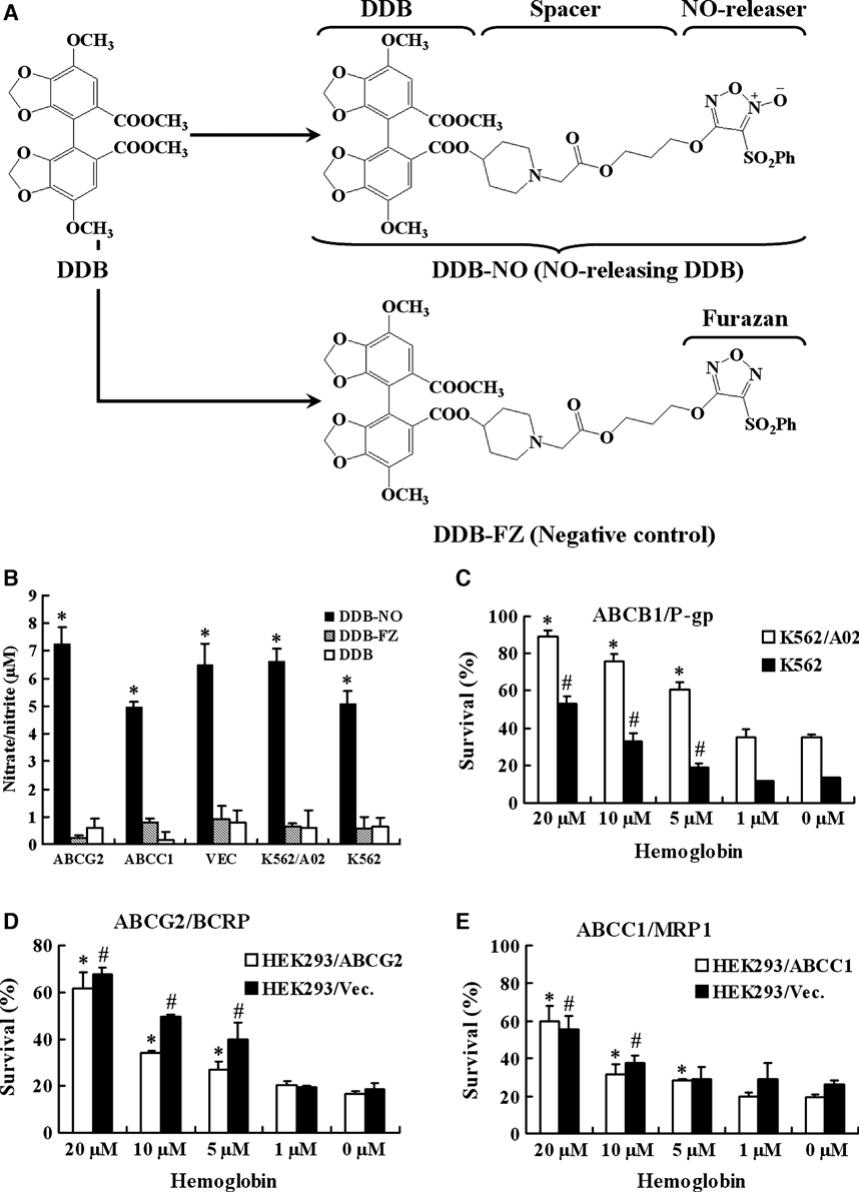
Nitric oxide released by DDB‐nitric oxide contributed to its inhibitory effect against sensitive and MDR cells. (**A**) The chemical structures of DDB, DDB‐nitric oxide (DDB‐NO) and DDB‐FZ. DDB‐nitric oxide consists of DDB linked to furoxan by a chemical spacer. DDB‐FZ (negative control) is structurally similar to DDB‐nitric oxide except no oxygen atom attached to the furazan. (**B**) Sensitive and three major MDR cell lines were treated with 100 μM DDB, DDB‐nitric oxide or DDB‐FZ for 240 min. and harvested for nitrate/nitrite Griess assay. Data are shown as mean values ± S.D. of the levels of nitrate/nitrite from three independent experiments. *P < 0.01 *versus* DDB control group. (**C**–**E**) Effect of DDB‐nitric oxide treated with haemoglobin against cancer cells. K562 and K562/A02 (**C**), or HEK293/Vec and HEK293/ABCG2 (**D**), or HEK293/Vec and HEK293/ABCC1 (**E**) were treated with 1 μM DDB‐nitric oxide in the absence or presence of different concentrations of haemoglobin for 24 hrs followed by SRB or MTS assays. The results are presented as survival rate (%) of cells relative to DMSO vehicle and shown as means ± S.D. of three independent experiments. *P < 0.05 *versus* control group (0 μM) in the MDR cells; #*P* < 0.05 *versus* control group (0 μM) in the sensitive cells. MDR, multidrug resistance.

In this study, we identified a compound, 4,4′‐Dimethoxy‐5,6,5′,6′‐dimethylenedioxy ‐2‐methoxycarbonyl‐2′‐{{N‐(2‐one‐2‐[3‐(3‐phenylsulfonyl‐1,2,5‐oxadiazole‐2‐oxide‐4‐)oxy] propoxy) ethyl}piperidyl‐4}oxycarbonyl biphenyl (named DDB‐nitric oxide or DDB‐NO, see Fig. 1A) that exhibited potential cytotoxicity against MDR cells as well as sensitive cancer cells at a comparatively low concentration. The preliminary mechanism studies suggest that the potent inhibitory effects of DDB‐nitric oxide is independent of the inhibition of three major drug transporters, but mostly relies on the promotion of mitochondrial tyrosine nitration and apoptosis, as well as the down‐regulation of HIF‐1α expression and AKT, ERK, and NF‐κB activation *via* high levels of nitric oxide release in MDR cells. Given that acquired MDR to nitric oxide donors is reportedly difficult to achieve and genetically unstable, compound like DDB‐nitric oxide may be a new type of therapeutic agent for better treatment of MDR tumours.

## Materials and methods

### Materials

Compounds DDB‐nitric oxide and DDB‐FZ with purity of >99% were synthesized in the State Key Laboratory of Natural Medicines in China Pharmaceutical University (Fig. S1). They were purified by column chromatography on silica gel 60 (200–300 mesh) or thin‐layer chromatography on silica gel 60 F254 plates. Subsequently, they were routinely analysed by IR, ^1^HNMR and ^13^CNMR, MS and HRMS. All of the compounds were dissolved in dimethyl sulfoxide (DMSO) at a concentration of 50 mM and stored at −20°C, in all experiments the final concentration of DMSO did not exceed more than 0.1% (v/v).

All electrophoresis reagents, protein concentration assay kits, and polyvinylidene difluoride membranes were purchased from Bio‐Rad (Hercules, CA, USA). Adriamycin and mitoxantrone were from Zhejiang Hisun Pharmaceutical Co., Ltd. (Taizhou, China), and Jiangsu Hansoh Pharmaceutical Co., Ltd. (Lianyungang, China) respectively. Rh‐123 and Sulforhodamine B (SRB) were obtained from Sigma‐Aldrich (St. Louis, MO, USA). MTS were purchased from Promega (Madison, WI, USA). Monoclonal antibody C‐219 against ABCB1, BXP‐21 against ABCG2 and 39B6 against 3‐NT were purchased from Abcam (Cambridge, MA, USA). MRPr1 against ABCC1 was from ARP American Research Products, Inc (Waltham, MA, USA). Anti‐PARP, Caspase‐3, Caspase‐9, AKT, p‐AKT, NF‐κB, p‐NF‐κB, ERK1/2 and p‐ERK1/2 antibodies were acquired from Cell Signaling Technology (Danvers, MA, USA). Anti‐HIF‐1α was purchased from BD Biosciences (San Jose, CA, USA). Real‐time PCR Master Mix was purchased from TOYOBO (Shanghai, China). DMSO, TRIZOL and G418 were purchased from Life Technologies (Grand Island, NY, USA). Nitric oxide assay kit, cell lysis buffer set for Western blot, haemoglobin, anti‐cytochrome c and Bax antibodies were purchased from Beyotime (Haimen, China). Cell culture medium RPMI 1640 and DMEM were purchased from HyClone (Logan, UT, USA). All other chemicals were obtained from commercial sources of analytical grade.

### Cell culture, treatments and lysate preparations

The human chronic myelogenous leukaemia cell line K562 and its drug‐selected cell line K562/A02 11 were kindly provided by Prof. Dongsheng Xiong (Institute of Hematology & Blood Diseases Hospital, CAMS & PUMC, China). HEK293‐transfected sublines HEK293/Vec 12, HEK293/ABCC1 13 and HEK293/ABCG2 14 were obtained from Prof. Jian‐ting Zhang (IU Simon Cancer Center, IN, USA). All cell lines from gifts were cultured as previously described and frozen into multiple aliquots. All cells were passaged for 4 months or less before the renewal from frozen, early‐passage stocks. Lysate preparation was performed as described previously 15.

### Western blot and FACS analyses

Western blot and Fluorescence Activated Cell Sorting (FACS) analyses of drug accumulation were performed exactly as we previously described 14, 15.

### Real time RT‐PCR

RNA extraction and real‐time RT‐PCR were performed as described previously 15. The sequences of ABCG2 primers are 5′‐TCATCAGCCTCGATATTCCATCT‐3′ (forward) and 5′‐GGCCCGTGGAACATAAGTCTT‐3′ (reverse). The sequences of ABCB1 primers are 5′‐CCCATCATTGCAATAGCAGG‐3′ (forward) and 5′‐GTTCAAACTTCTGCTCCTGA‐3′ (reverse). The sequences of ABCC1 primers are 5′‐CTTCCCACGGAGGAGTTT‐3′ (forward) and 5′‐GACCCAGACAAGGATGTTAGAGG‐3′ (reverse). The sequences of GAPDH primers are 5′‐CCGTCTAGAAAAACCTGCC‐3′ (forward) and 5′‐GCCAAATTCGTTGTC ATACC‐3′ (reverse). The standard curve and data analysis were produced using Bio‐Rad iQ5 software. The relative mRNA level treated with inhibitors was expressed as fold change in the control (in the presence of 0.1% DMSO).

### Cytotoxicity assay and nitric oxide‐eliminating experiment

Cytotoxicity was determined by using SRB and MTS assays as previously described 16, 17. The effect of inhibitors on drug resistance was determined by exposing cells to a low concentration (≤IC_20_) of anticancer drugs such as adriamycin and mitoxantrone in the absence or presence of DDB‐nitric oxide. The different non‐toxic concentrations of DDB‐nitric oxide (CH: 0.4 μM; CM: 0.2 μM; CL: 0.1 μM) were used to test the chemosensitization of MDR.

To further determine the contribution of nitric oxide released by DDB‐nitric oxide, the nitric oxide‐eliminating experiment was carried out by adding a nitric oxide scavenger, haemoglobin. Briefly, the cells were treated with different concentrations of haemoglobin (0, 1, 5, 10 or 20 μM) and 1 μM DDB‐nitric oxide for 24 hrs. Then the cell growths were determined by MTS or SRB as previously described. The cells treated with 0.1% DMSO (control) were considered as 100% survival, the survival rate (%) of different cells was expressed as percentage of the control and shown as means ± S.D. of three independent experiments.

### Nitrate/nitrite measurement *in vitro*

The levels of nitrate/nitrite produced by DDB‐nitric oxide, DDB‐FZ or DDB in the cells were determined by the nitrate/nitrite colorimetric assay kit as previously described 9, 10.

### Isolation of the Cytosolic and mitochondrial Fraction

Cells were washed twice with ice‐cold PBS, and the cell pellets were resuspended in 1 ml of ice‐cold buffer A (20 mM Hepes pH 7.5, 1.5 mM MgCl_2_, 10 mM KCl, 1 mM ethylenediaminetetraacetic acid (EDTA), 1 mM ethyleneglycol bis(2‐aminoethyl ether) tetraacetic acid (EGTA), 1 mM dithiothreitol (DTT), 0.1 mM PMSF) and 1× protease inhibitor cocktail (Roche), and containing 250 mM sucrose. The cells were homogenized 30 times in a Dounce homogenizer. After centrifugation three times at 1000 × g for 10 min. at 4°C, the supernatants were centrifuged at 16,000 × g for 30 min. at 4°C and the crude cytoplasmic (supernatant) and mitochondrial (precipitation) fractions were separated. Precipitations were washed two times with buffer A, then lysed in buffer B (150 mM NaCl, 1.0% NP40, 0.5% sodium deoxycholate, 0.1% SDS, 50 mM Tris pH 8.0, 5 mM EDTA, 1 mM EGTA, 5 mM NaF, 1 mM Na_3_VO_4_, 1× protease inhibitor cocktail) as mitochondrial fraction. For getting more pure cytosolic fraction, the resulting supernatants were centrifuged again at 70,000 × *g* for 1 hr at 4°C. Total protein concentrations in these fractions were determined using the Bio‐Rad protein assay with bovine serum albumin as the standard.

### Statistics

The IC_50_s of compounds were calculated by the software Prism 3.0 (GraphPad Software Inc., San Diego, CA, USA) for cell viability. All data are expressed as means ± S.D. for comparing treated groups with control group, a Student's *t*‐test was used. Values of *P* < 0.05 were considered significant.

## Results

### Cytotoxicity assay

We first compared DDB with DDB‐nitric oxide in terms of their growth inhibition in both parental sensitive and MDR cancer cell lines. To determine if the inhibitory effect of DDB‐nitric oxide was associated with nitric oxide release, we also synthesized another compound DDB‐FZ (see Fig. 1A) as a negative control which is structurally similar to DDB‐nitric oxide except no oxygen atom attached to the N‐2 atom of the furoxan ring, thereby not producing nitric oxide in all cells. As shown in Table 1, adriamycin exerted cytotoxicity against K562 and K562/A02 cancer cells with IC_50_ values of 0.59 and 24.47 μM, respectively, indicating K562/A02 cells are approximately 42‐fold resistant to adriamycin in comparison with the parental K562 cells. In sharp contrast, both the parental and MDR cell lines were almost equally inhibited by DDB‐nitric oxide with IC_50_ values of 0.78 and 0.59 μM respectively. DDB‐nitric oxide showed similar inhibitory activity as adriamycin in the K562 cells, but 20‐fold more potent than adriamycin in the K562/A02 cells that overexpress ABCB1. It was observed that DDB and DDB‐FZ had little inhibitory activity on both K562 and K562/A02 cells (IC_50_ >50 μM), suggesting that nitric oxide produced by furoxan moiety plays a crucial role for the inhibitory effects of DDB‐nitric oxide on K562 and K562/A02 cells. To directly test if DDB‐nitric oxide displays potency on the other major MDR cell lines, we performed similar studies using HEK293/ABCG2 and HEK293/ABCC1 which are ABCG2‐ and ABCC1‐transfected stable HEK293 cells respectively. As shown in Table 1, DDB‐nitric oxide significantly inhibited proliferation of sensitive HEK293/Vec (vector‐ transfected control) and MDR cells (IC_50_ <1 μM). Additionally, treatment with DDB‐nitric oxide (1.58 μM) promoted nearly 80% of inhibition in liver tumour HepG2 cells, while the same treatment only induced 10% of inhibition in liver normal LO2 cells (Fig. S2). The IC_50_ of DDB‐nitric oxide against HepG2 (0.99 μM) was 14‐fold less than LO2 (13.51 μM), indicating that DDB‐nitric oxide exhibited selective inhibitory effects on the growth of tumour cells compared to that of the normal cells. Therefore, these results indicated that DDB‐nitric oxide was very effective against both the parental‐sensitive and MDR cancer cells which overexpress three major ABC transporters at a comparatively low concentration.

**Table 1:** *In vitro* cytotoxicity of DDB‐nitric oxide, DDB‐FZ and DDB towards sensitive and multidrug resistance cell lines in comparison with adriamycin (ADR)

Compound	*In vitro* cytotoxicity (IC_50_, μM)				
	K562/A02	K562	HEK293/ABCG2	HEK293/ABCC1	HEK293/Vec.
DDB‐nitric oxide	0.59 ± 0.13	0.78 ± 0.33	0.62 ± 0.15	0.71 ± 0.21	0.69 ± 0.19
DDB‐FZ	>50	>50	>50	>50	>50
DDB	>100	>100	>100	>100	>100
ADR	24.47 ± 0.31	0.59 ± 0.48	1.95 ± 0.16	0.41 ± 0.12	0.04 ± 0.01

Values are mean ± S.D. from three independent experiments.

K562/A02 cells which overexpress ABCB1 were drug‐selected K562‐resistant subline cells; HEK293/Vec, HEK293/ABCG2 and HEK293/ABCC1 were vector‐, ABCG2‐, ABCC1‐ transfected stable cell lines respectively.

### Nitric oxide‐mediated cytotoxicity of DDB‐nitric oxide in the sensitive and MDR cells

To investigate if the strong cytotoxicity of DDB‐nitric oxide against resistant and sensitive cancer cells is associated with high levels of nitric oxide released by DDB‐nitric oxide, all of cells were exposed to DDB‐nitric oxide, DDB‐FZ or DDB and characterized by the Griess assay. As shown in Figure 1B, treatment with DDB‐nitric oxide promoted significant nitric oxide production in both sensitive and MDR cancer cells while only little nitric oxide was detected in the cells treated by DDB or DDB‐FZ, suggesting that the growth inhibition against both sensitive and MDR cancer cells may be attributable to nitric oxide produced by DDB‐nitric oxide.

To further determine the important role of nitric oxide released by DDB‐nitric oxide, all of the tested cells were treated with DDB‐nitric oxide in the absence or presence of haemoglobin, a known nitric oxide scavenger, followed by MTS or SRB assay. As shown in Figure 1C–E, treatment with DDB‐nitric oxide alone markedly inhibited the growth of different sensitive and MDR cancer cells, while the cells treated with various concentrations of haemoglobin significantly diminished the cytotoxicity of DDB‐nitric oxide in a dose‐dependent manner, supporting the above notion that nitric oxide produced by DDB‐nitric oxide plays a significant role for its inhibitory activity on both sensitive and MDR cells.

### Effect of DDB‐nitric oxide on three major MDR‐ABC transporters

**Figure 2:**
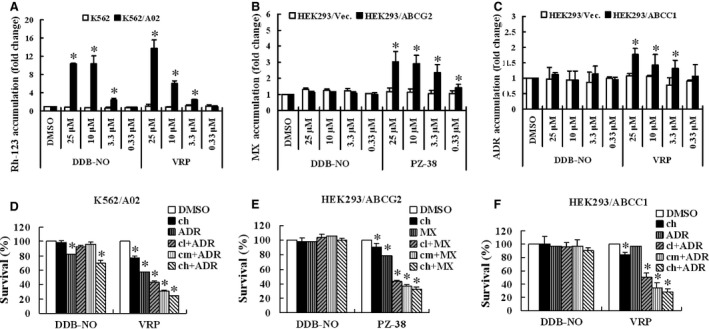
Effect of DDB‐nitric oxide on the intracellular substrate accumulation and chemo‐sensitization of drug resistance by ABCB1, ABCG2 and ABCC1. (**A**–**C**) Rh‐123 accumulation in K562 and K562/A02 cells (**A**) or mitoxantrone (MX) accumulation in HEK293/Vec and HEK293/ABCG2 cells (**B**) or adriamycin (ADR) accumulation in HEK293/Vec and HEK293/ABCC1 cells (**C**) following a 30 min. incubation in the absence or presence of different concentrations of DDB‐nitric oxide, verapamil (VRP) or PZ‐38. PZ‐38 was used as an ABCG2 inhibitor control, while VRP was used as a positive control of ABCB1 and ABCC1. The data are means ± S.D. from three independent experiments. (**D**–**F**) K562/A02 (**D**) or HEK293/ABCG2 (**E**) or HEK293/ABCC1 (**F**) cells were treated with or without IC_20_ of adriamycin or mitoxantrone in the absence or presence of different concentrations of DDB‐nitric oxide (CH: 0.4 μM; CM: 0.2 μM; CL: 0.1 μM) followed by SRB or MTS assay. PZ‐38 and VRP were used as inhibitor controls of ABCG2, ABCB1 and ABCC1 (CH: 2.5 μM; CM: 1.25 μM; CL: 0.625 μM for PZ‐38 and VRP). The results are presented as survival rate (%) of cells relative to DMSO vehicle and shown as means ± S.D. of three independent experiments. *P < 0.05 *versus* DMSO control group.

**Figure 3:**
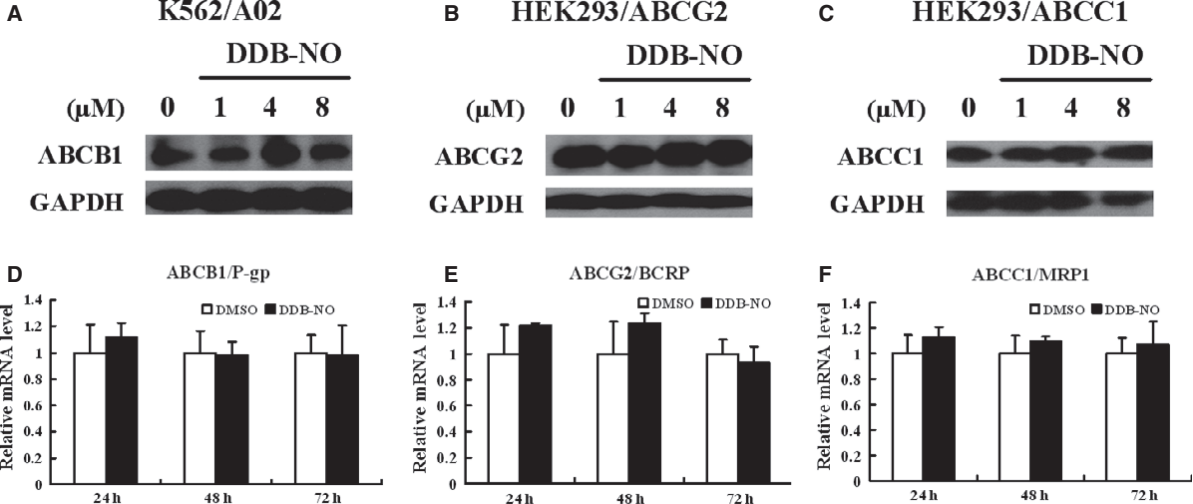
Effect of DDB‐nitric oxide on the protein and mRNA expression level of ABCB1, ABCG2 and ABCC1. (**A**–**C**) K562/A02, HEK293/ABCG2 or HEK293/ABCC1 cells were treated with 0, 1, 4 or 8 μM of DDB‐nitric oxide for 24 hrs and harvested for Western blot analysis of ABCB1 (**A**), ABCG2 (**B**) and ABCC1 (**C**), respectively. GAPDH was used as a loading control. The data are representative of three independent experiments. (**D**–**F**) K562/A02 (**D**) or HEK293/ABCG2 (**E**) or HEK293/ABCC1 (**F**) cells were treated with DMSO vehicle or IC_50_ of DDB‐nitric oxide for various times and harvested for RNA preparation and real‐time RT‐PCR analysis. Data shown are mean ± S.D. from three independent experiments.

Given that inhibition of MDR‐ABC transporters efflux is one of the effective strategies to overcome MDR, we suggested that DDB‐nitric oxide might exert strong cytotoxicity on different MDR cells by inhibiting the functions or expressions of MDR‐ABC transporters. To test this hypothesis, firstly, we determined the effects of DDB‐nitric oxide on functions of MDR‐ABC transporters using flow cytometry. As shown in Figure 2A, DDB‐nitric oxide enhanced rhodamine‐123 (Rh‐123, substrate of ABCB1) accumulation in a dose‐dependent manner in K562/A02 but not in the parental K562 cells that do not overexpress ABCB1, suggesting ABCB1‐mediated drug efflux has been inhibited. However, treatment with DDB‐nitric oxide at a low dose of 0.33 μM which is near to its value of IC_50_ restored only little intracellular Rh‐123 level in the K562/A02 cells, indicating that it displayed strong cytotoxicity not by inhibiting ABCB1 function. We also found no effect of DDB‐nitric oxide on the drug efflux activity of ABCG2 and ABCC1 respectively (Fig. 2B and C). Secondly, we determined if DDB‐nitric oxide has the ability to sensitize ABC transporter‐mediated MDR cells to some known anticancer drugs, such as mitoxantrone (MX) and adriamycin (ADR) which were used as the model substrates of ABCG2, ABCB1 and ABCC1. As shown in Figure 2D–F, DDB‐nitric oxide at non‐toxic dose did not significantly alter the cytotoxicity of mitoxantrone and adriamycin in K562/A02, HEK293/ABCG2 and HEK293/ABCC1 MDR cells, suggesting that DDB‐nitric oxide do not chemo‐sensitize MDR mediated by ABCB1, ABCG2 and ABCC1 transporters. Thirdly, we determined whether DDB‐nitric oxide can reduce the protein expression level of three major MDR‐ABC transporters in all of test MDR cells. As shown in Figure 3A–C, the expressions of ABCB1, ABCG2 and ABCC1 proteins was not affected by treating different MDR cells with various doses of DDB‐nitric oxide for 24 hrs, compared with vehicle control (0.1% DMSO). As can be seen in Figure 3D–F, no significant changes were observed in the mRNA expression level of ABCB1, ABCG2 and ABCC1 by real‐time RT‐PCR analyses. These results suggest that DDB‐nitric oxide have no effects on the protein and mRNA expression levels of three major MDR‐ABC transporters. Taken together, we concluded that DDB‐nitric oxide exerted its cytotoxicity on those MDR cells not by inhibiting functions and expressions of MDR‐ABC transporters.

Since efflux of drug by ABCB1 normally requires energy from ATP hydrolysis by the ATPase, which is generally stimulated in the presence of ABCB1 substrate, some identified reversal agents such as verapamil stimulate the basal ATPase activity, thereby behaving like a competitive inhibitor for the pump 18. Considering high concentrations of DDB‐nitric oxide exerted MDR reversal effect in the K562/A02 cells, we next examined the effect of DDB‐nitric oxide or DDB on ABCB1 ATPase activity according to a previously described method 19. As shown in Table S1, the luminescence value of each sample represented its ATP level, which is negatively correlated with the activity of ABCB1 ATPase. Verapamil (stimulator control) caused a significant increase in the activity of ABCB1 ATPase compared with DMSO group, while DDB‐nitric oxide, DDB or Na_3_VO_4_ (inhibitor control) showed no stimulation of the ABCB1 ATPase activity, indicating that DDB‐nitric oxide was not a substrate of ABCB1.

### Effect of DDB‐nitric oxide on mitochondrial apoptotic pathway protein

**Figure 4:**
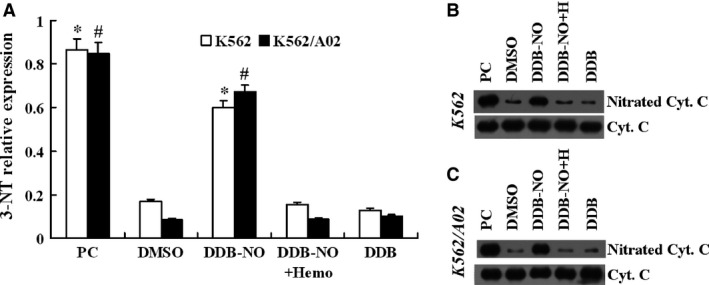
Effect of DDB‐nitric oxide on mitochondrial protein 3‐nitrotyrosine (3‐NT) formation. K562 or K562/A02 cells were treated with DMSO vehicle, 10 μM of DDB or DDB‐nitric oxide in the absence or presence of 20 μM haemoglobin (Haemo or H) for 24 hrs and their mitochondrial proteins were extracted for Western blot analysis of cytochrome c (Cyt c) related 3‐NT. Tyrosine‐nitrated Cyt c was prepared as described and used as positive control (PC). (**A**) Data shown are mean ± S.D. from 3 independent experiments. *P < 0.01 *versus* DMSO group in the K562 cells; #*P* < 0.01 *versus* DMSO group in the K562/A02 cells. The data are representative of three independent experiments in the K562 (**B**) and K562/A02 (**C**) cells.

It has been previously reported that high levels of nitric oxide can lead to the formation of reactive nitrogen species (RNS) such as peroxynitrite in mitochondria 20. Generally, peroxynitrite can covalently modify tyrosine residues in mitochondrial proteins to form 3‐nitrotyrosine (3‐NT). Therefore, determination of 3‐NT has been widely used as a reliable peroxynitrite biomarker 21. As mentioned above, the high levels of nitric oxide contributed to cytotoxicity of DDB‐nitric oxide, thus we examined whether sensitive and MDR cancer cells treated with DDB‐nitric oxide could result in an increase in nitration of the mitochondrial cytochrome c (Cyt c) protein. In view of recent trends in the research of three major MDR‐ABC transporters, ABCB1 stands out by conferring the strongest resistance to the widest variety of anticancer drugs, and shows high correlation with clinical MDR and poor outcome. K562 and K562/A02 cells were selected as representative object and treated with DDB or DDB‐nitric oxide for 24 hrs, and then mitochondrial proteins were extracted and harvested for Western blot analysis of 3‐NT. As shown in Figure 4, DDB‐nitric oxide produced a significant increase in mitochondrial Cyt c‐related 3‐NT level in both K562 and K562/A02 cells, similar to the effect of treatment with peroxynitrite (positive control) on Cyt c protein. However, addition of haemoglobin, a nitric oxide scavenger, decreased the levels of the 3‐NT in the presence of DDB‐nitric oxide in all tested cells. We also found no effect of DDB, a lead compound of DDB‐nitric oxide, on the formation of the 3‐NT. Consistent with the fact that high cytotoxic activity of DDB‐nitric oxide decreased in the presence of haemoglobin in sensitive and MDR cancer cells, we concluded that high levels of nitric oxide released by DDB‐nitric oxide generated a large amount of peroxynitrite in mitochondria, which should be responsible for, at least in part, the cytotoxicity of DDB‐nitric oxide.

**Figure 5:**
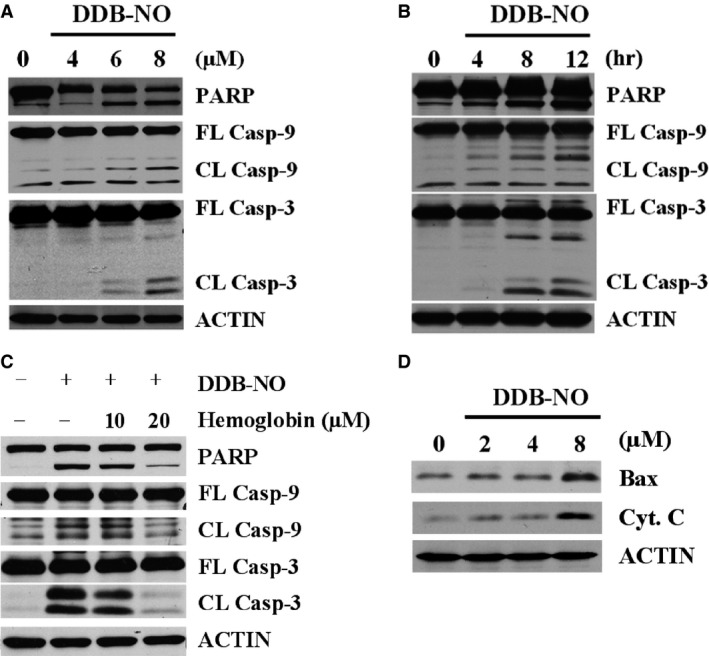
Effect of DDB‐nitric oxide on mitochondrial apoptotic pathway in ABCB1‐overexpressing cells. (**A**) K562/A02 cells were treated with 0, 4, 6 or 8 μM of DDB‐nitric oxide for 12 hrs, or (**B**) K562/A02 cells were treated with 8 μM DDB‐nitric oxide for 4, 8, 12 hrs, or (**C**) K562/A02 cells were treated with 8 μM DDB‐nitric oxide in the absence or presence of different concentrations of haemoglobin for 12 hrs, and then harvested for Western blot analysis of cleavage of PARP, caspase‐9 and caspase‐3. (**D**) K562/A02 cells were treated with 0, 2, 4 or 8 μM of DDB‐nitric oxide for 12 hrs, followed by immunoblotting for Bax and cytochrome c (Cyt. C). Actin was used as a loading control. The data are representative of three independent experiments.

To determine whether DDB‐nitric oxide induces cleavage of PARP as well as caspase‐9 and caspase‐3 in the mitochondrial apoptotic pathway, K562/A02 cells were exposed to DDB‐nitric oxide and harvested for Western blotting. As shown in Figure 5A and B, DDB‐nitric oxide induced significant PARP, caspase‐9 and caspase‐3 cleavage in K562/A02 MDR cells in a dose‐ and time‐dependent manner. Addition of haemoglobin to scavenge nitric oxide significantly diminished the effect of DDB‐nitric oxide on PARP, caspase‐9 and caspase‐3 cleavage in K562/A02 cells (Fig. 5C), supporting the argument that nitric oxide released by DDB‐nitric oxide promotes apoptosis by the activation of mitochondrial pathway in the MDR cells. To further examine whether the effect of DDB‐nitric oxide is associated with the mitochondrial pathway, K562/A02 cells were treated with various doses of DDB‐nitric oxide for 12 hrs, followed by immunoblotting for Bax and Cyt c. The dose–response experiments showed that the activation of Bax by DDB‐nitric oxide induced Cyt c release (Fig. 5D) followed by caspase‐9, caspase‐3 and PARP cleavage, suggesting that the involvement of the mitochondrial pathway is mostly associated with the cytotoxicity of DDB‐nitric oxide.

### Effects of DDB‐nitric oxide on the expression of HIF‐1α and regulatory signalling pathway in MDR cells

**Figure 6:**
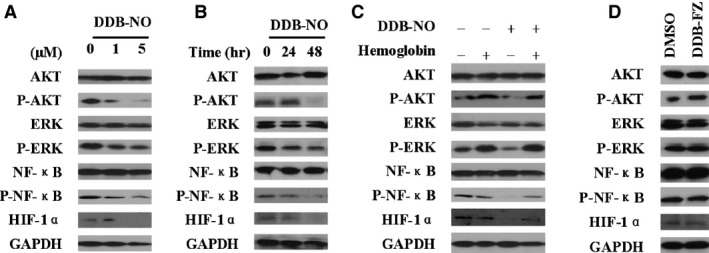
Effect of DDB‐nitric oxide on AKT, ERK, NF‐κB and HIF‐1α in ABCB1‐overexpressing cells. (**A**) K562/A02 cells were treated with 0, 1 or 5 μM of DDB‐nitric oxide for 24 hrs, or (**B**) K562/A02 cells were treated with 1 μM DDB‐nitric oxide for 24 or 48 hrs, or (**C**) K562/A02 cells were treated with 4 μM DDB‐nitric oxide in the absence or presence of 20 μM haemoglobin for 24 hrs, or (**D**) K562/A02 cells were treated with 5 μM DDB‐PZ for 24 hrs, and then harvested for Western blot analysis of HIF‐1α, NF‐κB, AKT and ERK. GAPDH was used as a loading control. The data are representative of three independent experiments.

Multidrug resistance is a major problem in cancer chemotherapy. It has also been reported previously that potential resistant mechanism of actions include the activation of the Ras/Raf/MEK/ERK and PI3K/AKT pathway as well as key transcription factors such as NF‐κB and HIF‐1α that play a central role in proliferation and survival of cancer cells 7. To investigate if the effects of DDB‐nitric oxide on MDR cells *via* the inhibition of these key signalling pathways, we examined the levels of total HIF‐1α and phosphorylated forms of AKT, ERK, NF‐κB after treatment with DDB‐nitric oxide in the resistant cells. As shown in Figure 6A and B, DDB‐nitric oxide significantly down‐regulated p‐AKT, p‐ERK and p‐NF‐κB in a dose‐ and time‐dependent manner in K562/A02 cells, although the total AKT, ERK and NF‐κB levels remained unchanged. Western blotting also showed that the expression of HIF‐1α was affected by treating K562/A02 cells with DDB‐nitric oxide, compared with vehicle control (0.1% DMSO, 0 hr). As shown in Figure 6C, addition of haemoglobin to scavenge nitric oxide effectively restored the total levels of HIF‐1α expression and AKT, ERK, NF‐κB activation in K562/A02 cell lines treated with DDB‐nitric oxide, supporting the above notion that nitric oxide released by DDB‐nitric oxide plays an important role in MDR cell lines. Figure 6D also showed that negative compound DDB‐FZ at the same concentration did not significantly alter the key target protein activities in the signalling pathway, compared with vehicle control. Taken together, these results indicated that the suppression of HIF‐1α expression and AKT, ERK, NF‐κB phosphorylation may be most likely associated with the high levels of nitric oxide released by DDB‐nitric oxide in MDR cells.

## Discussion

Multidrug resistance is often associated with the overexpression of three major ABC transporters, including ABCB1, ABCG2 and ABCC1. Recent reports suggest that the development of MDR not only actively transports a wide range of cytotoxic drugs across MDR‐ABC transporters but is also a complex interaction between a number of important cellular pathways that help cancer cells to sustain malignancy and evade the cytotoxic effects of anticancer drugs 22. Therefore, nitric oxide donors appear to be a new class of anticancer therapeutics for satisfying the above conditions. In this study, we compared the efficacy of the parental and nitric oxide‐modified drugs against sensitive and MDR tumour cells which overexpress three major MDR‐ABC transporters. DDB‐nitric oxide is a nitric oxide‐releasing derivative of DDB. Its mechanism of action appears to be in two modes by inducing mitochondrial tyrosine nitration and apoptosis as well as by down‐regulating HIF‐1α expression and AKT, ERK and NF‐κB activation *via* high levels of nitric oxide release in MDR cells.

Various types of nitric oxide donor or mimetics, such as s‐nitrosopenicillamin and nitroglycerine, have been reported to reverse the resistance by inhibiting drug efflux mediated by MDR‐ABC transporters *in vitro* and *in vivo* 23, 24. Interestingly, Zou *et al*. reported that some furoxan‐based nitric oxide‐releasing derivatives of tetrahydroisoquinoline exhibited potent MDR reversal activities on K562/A02 cell line, suggesting that cytotoxic activities increased as the increasing concentration of nitric oxide production 25. In our study, DDB‐nitric oxide has the same nitric oxide‐releasing moiety (furoxan) but different scaffold (DDB) and spacer. Although DDB‐nitric oxide inhibited ABCB1‐mediated drug efflux in a dose‐dependent manner, its strong cytotoxicity was not *via* inhibiting ABCB1 function at the dose which is close to its value of IC_50_. DDB‐nitric oxide showed no differences in potency against the parental sensitive and MDR cell lines. Treatment with DDB‐nitric oxide promoted high levels of nitric oxide production (more than 5 μM) in our study compared with the amount of 1.5 μM nitric oxide release by their furoxan‐based nitric oxide‐donating derivatives of tetrahydroisoquinoline in the same K562/A02 cell line. These data suggest that the difference trends between cell growth inhibitory and reversal effects of compounds on resistant cells may be attributed to the different amounts of nitric oxide production. However, the precise structure‐activity relationships remain to be elucidated when more furoxan‐based nitric oxide‐releasing donors with different parent structures and chemical spacers will be available in the future.

Mitochondria are the potential targets for a new class of anticancer therapeutics against the resistant cancer cells 26. It is increasingly recognized that nitric oxide‐induced peroxynitrite formation could induce apoptosis in cancer cells. As a relevant biomarker of peroxynitrite, the increased levels of mitochondrial protein 3‐NT represent high levels of nitric oxide release. In our study, DDB‐nitric oxide significantly promoted generation of mitochondrial Cyt c‐related 3‐NT and could be abolished by nitric oxide scavenger in both sensitive and MDR cancer cells, suggesting that the effect of DDB‐nitric oxide might be attributed to the high levels of nitric oxide release. Next, we determined whether the DDB‐nitric oxide‐induced apoptosis was associated with the apoptotic proteins in the mitochondria pathway. Our results showed that treatment with DDB‐nitric oxide resulted in activation of Bax, accompanied by release of Cyt c followed by cleavage of caspase‐9, caspase‐3 and PARP, demonstrating that apoptosis *via* the mitochondrial pathway is involved in the cytotoxicity of DDB‐nitric oxide in MDR cancer cells. Recently, Hoffman *et al*. developed cancer cells expressing red fluorescent protein in the cytoplasm and green fluorescent protein linked to histone H2B expressed in the nucleus for early detection of apoptosis in the rapidly screen of cancer chemotherapy agents 27, 28, 29, 30. It is better suited for detecting the real‐time cellular apoptosis than caspase and other apoptosis assays which require multiple steps. Clearly, further studies on the real‐time cellular assay for apoptosis will be needed to elucidate cytoplasmic and nuclear shape changes and nuclear fragment in the dual‐colour MDR cells treated with nitric oxide‐releasing agents.

Based on the knowledge of clinical study, it appears that drug resistance might be involved with multiple factors in enhanced cell survival when treated with anticancer agents 22. It was proposed that controlling the key signalling pathways related to resistance mechanisms could be the most effective way to eliminate MDR in cancer patients. It has also been reported previously that resistance to some anticancer drugs in cancer cells can be decreased by inhibiting the Ras/Raf/MEK/ERK and PI3K/AKT pathways 31, 32 and key transcription factors, such as NF‐κB and HIF‐1α 7. Our results suggested that DDB‐nitric oxide significantly down‐regulated HIF‐1α, p‐AKT, p‐ERK and p‐NF‐κB in a dose‐ and time‐dependent manner in MDR cells, although the total AKT, ERK and NF‐κB levels remained unchanged. Addition of nitric oxide scavenger restored the proliferative effect of HIF‐1α expression and NF‐κB, AKT and ERK activation, suggesting the important role of nitric oxide in the inhibitions of intracellular key signalling pathways. Since recent reports have revealed that increase in HIF‐1α was paralleled by an increase of carbonic anhydrase type XII (CAXII) and ABCB1 during the acquisition of chemoresistance 33, more studies are needed to evaluate the effect of DDB‐nitric oxide on CAXII activity in MDR cells. On the whole, these data showed that the high cytotoxicity of DDB‐nitric oxide in MDR cell lines, at least in part, correlated with the inhibitions of HIF‐1α expression and NF‐κB, AKT, ERK phosphorylation *via* high levels of nitric oxide release.

Recent publications showed some special characteristics of nitric oxide‐donors. Hutchens *et al*. reported that HL60 cells which were exposed to a nitric oxide‐releasing prodrug (PABA/ nitric oxide) for 6 months, only exhibited 1.9‐fold resistance to the drug, and the sensitivity of resistant cells was reverted within 3 weeks after removing PABA/nitric oxide from the growth medium 8, indicating that acquired drug resistance to nitric oxide‐donors is difficult to achieve and genetically unstable. Interestingly, Rothweiler *et al*., at the first time, investigated the potential role of nitric oxide‐modified saquinavir derivative (Saq‐nitric oxide) as anticancer drug in cell lines overexpressing three major MDR‐ABC transporters 34. Saq‐nitric oxide exerted anticancer effects in a panel of human MDR cancer cell lines with IC_50_ value ranged from 6 to 14 μM. Non‐toxic concentrations Saq‐nitric oxide caused sensitization of ABCB1‐, ABCC1‐ or ABCG2‐overexpressing cancer cells to chemotherapy. However, it is less obvious whether Saq‐nitric oxide acts as anticancer drug *via* targeting the key signalling pathway. Our study showed that DDB‐nitric oxide exhibited no differences in potency against the parental and MDR cell lines. Moreover, DDB‐nitric oxide inhibited a number of key signalling pathways implicated in cancer drug resistance and tumour proliferation, probably as a result of producing much higher levels of nitric oxide in tumour cells. However, it is unknown if DDB‐nitric oxide has a broad antitumour spectrum *in vitro* and is effective on MDR tumours *in vivo*, although our previous results revealed that a variety of furoxan‐based nitric oxide donors exhibited selective antitumour activity *in vitro* and *in vivo* 10, 35, 36. Regardless, these possibilities need to be evaluated in the future studies using animal models.

Targeting the cell cycle becomes a common knowledge for the discovery and development of novel anticancer agent, especially in the cell cycle progression and cell cycle‐associated cell death. Recently, the combination of Fluorescence Ubiquitin Cell Cycle Indicator (FUCCI) imaging and 3‐dimensional Gelfoam^®^ histoculture has been developed, which label individual G1 phase nuclei red and those protein in S/G2/M phases green 37, 38. Fluorescence Ubiquitin Cell Cycle Indicator cells change colour from red to green as they transit from G1 to S/G2/M phases. It is obvious to us that the cell cycle phase distribution of cancer cells in G0/G1 phase in Gelfoam^®^ histoculture is highly similar to *in vivo* tumours by FUCCI imaging, differently than cancer cells in 2D culture. Longitudinal real‐time tracking also demonstrated that cytotoxic drugs killed only proliferating cancer cells in S/G2/M phases and had little effect on resistant quiescent cancer cells in G0/G1 phase, where cancer chemotherapy is not valid 39, 40. Therefore, the application of FUCCI technology provide a more effective way of evaluating anticancer agents that target quiescent cancer cells in G0/G1 phase which resistant to cytotoxic drug already used in the clinic. Further studies of visualizing real‐time cell cycle dynamics of MDR cancer cells treated with nitric oxide‐releasing agents throughout a live tumour may help address the potential effect of DDB‐nitric oxide.

In conclusion, unlike any previously reported nitric oxide‐donors as MDR reversal agents, DDB‐nitric oxide showed significantly potent growth inhibitory effects on sensitive and MDR cell lines. Apparently, the production of higher levels of nitric oxide by DDB‐nitric oxide in all tested tumour cells mainly contributed to its potent inhibitory effect which may be independent of the inhibition of three major drug transporters. Additionally, the two modes of actions make DDB‐nitric oxide a very interesting and promising anticancer agent for further exploitation. In the first mode, DDB‐nitric oxide appears to induce mitochondrial tyrosine nitration and apoptosis by release of Bax and cytochrome c followed by caspase‐9, caspase‐3 and PARP cleavages. In the second mode, DDB‐nitric oxide also inhibits a number of key signalling pathways implicated in cancer drug resistance and tumour proliferation. Addition of a typical nitric oxide scavenger significantly attenuated the effects of DDB‐nitric oxide, suggesting that its cytotoxicity may be because of the higher levels of nitric oxide release in MDR cancer cells. Given that acquired MDR to nitric oxide donors was difficult to achieve and genetically unstable, compounds like DDB‐nitric oxide may be a new type of therapeutic agent for better treatment of MDR tumours.

## Conflict of interest

The authors declare no conflicts of interest.

## Supporting information


**Figure S1** Synthetic route of compounds of DDB‐nitric oxide and DDB‐FZ.
**Figure S2** Inhibition of DDB‐nitric oxide on the proliferation of HepG2 and LO2 cells.
**Table S1** Effect of DDB‐nitric oxide on ABCB1 ATPase activity.
