# Supplementary material for: Anticancer efficacy of a nitric oxide‐modified derivative of bifendate against multidrug‐resistant cancer cells
Source: J Cell Mol Med. 2016 Feb 10;20(6):1095–105. doi: 10.1111/jcmm.12796 (PMC4882976; doi:10.1111/jcmm.12796)
Supplement: Supplementary file 1 — Figure S1 Synthetic route of compounds of DDB‐nitric oxide and DDB‐FZ. Figure S2 Inhibition of DDB‐nitric oxide on the proliferation of HepG2 and LO2 cells. Table S1 Effect of DDB‐nitric oxide on ABCB1 ATPase activity. [file JCMM-20-1095-s001.doc]

**Figure S1. Synthetic route of compounds of DDB-NO and DDB-FZ.** Reagents and conditions: (a) diol, THF, 30% NaOH, rt, 4 h; (b) chloroacetic acid, EDCI, DMAP, CH_2_Cl_2_, rt, 6 h; (c) 4-hydroxypiperidine, dioxane, DMAP, 40 °C, 6-8 h; (d) oxalyl chloride, CH_2_Cl_2_, rt, 1h; (e) **4**, CH_2_Cl_2_, DMAP, rt, 8-12h; (f) P(OCH_3_)_3_, reflux, 10 h.

**
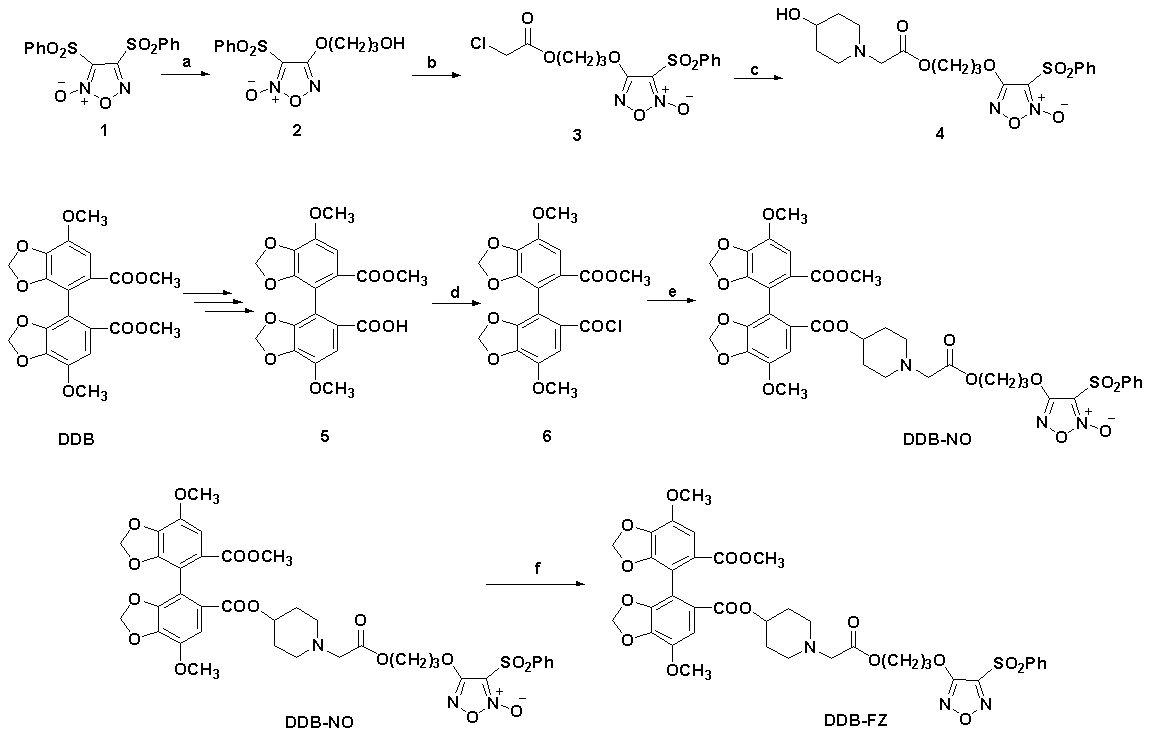
**

**Figure S2. Inhibition of DDB-NO on the proliferation of** **HepG2 and LO2 cells.** HepG2 and LO2 cells were treated with the indicated concentrations of DDB-NO for 72 h. The inhibitory effect on the proliferation of DDB-NO was determined by SRB assay as previously described. The data are representative of 3 independent experiments.

**
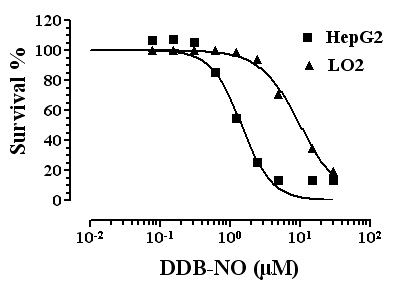
**

**Table S1** Effect of DDB-NO on ABCB1 ATPase activity.

| Compound | Concentration (μM) | Luminescence (relative light units)# |
| --- | --- | --- |
| DMSO | 0 | 216,508 ± 7426 |
| Verapamil | 200 | 179,086 ± 3939* |
| Na_3_VO_4_ | 200 | 242,242 ± 1851* |
| DDB | 40 | 252,298 ± 195* |
| DDB-NO | 1 | 246,255 ± 3812* |
| DDB-NO | 40 | 279,806 ± 1390** |

# Relative light units represent the level of ATP in the sample, exhibiting a negative relationship with activity of ABCB1 ATPase. Data are expressed as mean ± SD of three separate experiments. **P*< 0.05, ** *P*< 0.01 *vs.* DMSO group, determined by Student's *t* test.

The ATPase activity of ABCB1 was determined using crude membranes prepared from K562/A02 cells and the luminescent ATP detection kit (Pgp-Glo^TM^ Assay System without P-glycoprotein, Promega, USA) with some modification. Crude cell membranes were prepared in exactly the same way as previously described [ref.19 in the article] and final membranes were resuspended in 10mM Tris-HCl (pH 7.5). The effect of DDB-NO on ABCCB1 ATPase activity was then determined by the the luminescent ATP detection kit according to the manufacturer's instruction. Briefly, DDB-NO at 1 or 40 μM in buffer solution were first incubated with 0.6 mg/ml crude membranes and 5 mM MgATP at 37℃ for 40 min. Luminescent was initiated by ATP detection buffer, and then the remaining ATP was detected after incubated at room temperature for 40 min to allow luminescent signal to develop. Sodium vanadate (Na_3_VO_4_) and DDB were used as inhibitor control, whereas Verapamil was used as substrate control, respectively. Changes of RLU (relative light units) reflect the ATP level in the sample, which is negatively correlated with the activity of ABCB1 ATPase and was detected in a luminometer (Perkin Elmer TD-20, USA).
